# A Mathematical Model of the Enhanced Permeability and Retention Effect for Liposome Transport in Solid Tumors

**DOI:** 10.1371/journal.pone.0081157

**Published:** 2013-12-02

**Authors:** Shawn Stapleton, Michael Milosevic, Christine Allen, Jinzi Zheng, Michael Dunne, Ivan Yeung, David A. Jaffray

**Affiliations:** 1 Department of Medical Biophysics, University of Toronto, Ontario, Canada; 2 STTARR Innovation Centre, Princess Margaret Cancer Centre, Toronto, Ontario, Canada; 3 Radiation Medicine Program, Princess Margaret Cancer Centre, Toronto, Ontario, Canada; 4 Department of Radiation Oncology, University of Toronto, Toronto, Ontario, Canada; 5 Leslie Dan Faculty of Pharmacy, University of Toronto, Toronto, Ontario, Canada; 6 Techna Institute, University Health Network, Toronto, Ontario, Canada; National Health Research Institutes, Taiwan

## Abstract

The discovery of the enhanced permeability and retention (EPR) effect has resulted in the development of nanomedicines, including liposome-based formulations of drugs, as cancer therapies. The use of liposomes has resulted in substantial increases in accumulation of drugs in solid tumors; yet, significant improvements in therapeutic efficacy have yet to be achieved. Imaging of the tumor accumulation of liposomes has revealed that this poor or variable performance is in part due to heterogeneous inter-subject and intra-tumoral liposome accumulation, which occurs as a result of an abnormal transport microenvironment. A mathematical model that relates liposome accumulation to the underlying transport properties in solid tumors could provide insight into inter and intra-tumoral variations in the EPR effect. In this paper, we present a theoretical framework to describe liposome transport in solid tumors. The mathematical model is based on biophysical transport equations that describe pressure driven fluid flow across blood vessels and through the tumor interstitium. The model was validated by direct comparison with computed tomography measurements of tumor accumulation of liposomes in three preclinical tumor models. The mathematical model was fit to liposome accumulation curves producing predictions of transport parameters that reflect the tumor microenvironment. Notably, all fits had a high coefficient of determination and predictions of interstitial fluid pressure agreed with previously published independent measurements made in the same tumor type. Furthermore, it was demonstrated that the model attributed inter-subject heterogeneity in liposome accumulation to variations in peak interstitial fluid pressure. These findings highlight the relationship between transvascular and interstitial flow dynamics and variations in the EPR effect. In conclusion, we have presented a theoretical framework that predicts inter-subject and intra-tumoral variations in the EPR effect based on fundamental properties of the tumor microenvironment and forms the basis for transport modeling of liposome drug delivery.

## Introduction

The discovery of the enhanced permeability and retention (EPR) effect in solid tumors has led to the development of a wide range of nanomedicines, including liposomes, for cancer therapy [1]. The EPR effect describes the preferential accumulation of nanoparticles at tumor sites due to leaky vasculature (i.e. enhanced permeation) and impaired lymphatic drainage (i.e. enhanced retention), in comparison to normal tissue. Nano-sized delivery systems have been shown to result in significant increases in tumor accumulation of drugs in comparison to that achieved following administration of free drug [2]. Yet, despite demonstrating substantial accumulation of drug in many pre-clinical and human tumors [2]–[4], clinically approved liposome formulations, such as Doxil®/Caelyx® (pegylated liposomal doxorubicin) and Myocet® (unpegylated liposomal doxorubicin), have only resulted in a modest increase in anti-tumor efficacy relative to the standard of care [5]–[9]. Major limitations of liposome-based drug delivery are: (1) variability in the EPR effect and therefore, total tumor accumulation [4]; (2) limited tumor penetration [10]; and (3) slow or limited release of hydrophilic/amphiphilic drugs [11], [12]. While it is clear that the poor performance has been linked to a number of factors, one of the most significant is the inability to achieve consistent inter-subject and intra-tumoral accumulation of liposomes [4], [10], [13], [14].

Heterogeneity in liposome accumulation implies the presence of inter-subject and intra-tumoral variations in the EPR effect. Several studies have indicated variations in EPR are driven by abnormalities in the tumor microenvironment, including heterogeneity in vascular permeability, and elevated interstitial fluid pressure (IFP) [10], [13], [15]–[18]. Medical imaging has emerged as an important method to non-invasively detect liposome accumulation *in vivo*, which in turn provides direct visualization of variations in the EPR effect. In the clinical setting significant inter-subject variations have been observed using whole body gamma camera imaging of ^111^In labeled liposome accumulation in many different solid tumors [4], [19], [20]. Pre-clinical imaging using high resolution computed tomography (CT) has shown significant heterogeneity in the intra-tumoral distribution of liposomes with larger tumors exhibiting predominantly peripheral accumulation [21]–[23]. Beyond visualization of inter-subject and intra-tumoral variations in liposome accumulation, imaging the spatio-temporal distribution of liposomes may also provide information about the underlying transport properties of solid tumors that affect accumulation. This can be accomplished by fitting measurements of liposome accumulation with a biophysical mathematical model that describes liposome transport. The combination of imaging and mathematical modeling of liposome transport then provides the ability to quantitatively relate inter-subject and intra-tumoral variations in the EPR effect to properties of the tumor microenvironment.

Several modeling approaches, including physiological based pharmacokinetic (PBPK), as well as spatially distributed diffusion-convection models, have been proposed to describe the transport of nano-sized vehicles to solid tumors [24]–[27]. PBPK models provide a simple framework to describe the accumulation of macromolecules and nanoparticles in tumors, but are limited in their ability to describe transport properties and neglect intra-tumoral distribution [24], [27]. In contrast, spatially distributed diffusion-convection models provide an explicit biophysical framework to describe the transport of fluid, macromolecules and nanoparticles across blood vessels and through the interstitial space of solid tumors using parameters that reflect nanoparticle and tumor microenvironment properties. [17], [24], [27].

Therefore the aim of this work was to develop and validate a spatially distributed biophysical transport model that can quantitatively relate measurements of liposome accumulation to inter-subject and intra-tumoral variations in the EPR effect caused by the underlying transport dynamics in a solid tumor. We test the transport model using an image-based approach that allows for a direct comparison of the predicted and measured accumulation of a CT liposome contrast agent in solid tumors. The contrast agent is comprised of liposomes that encapsulate iohexol, and has been shown to accumulate in solid tumors via the EPR effect [21]–[23]. The transport model, hereafter referred to as the ‘Intra-Tumor Transport Model’ (ITTM), was developed to describe the inter-subject and intra-tumoral transport of liposomes. However, while the model has been developed to include spatio-temporal variations in liposome accumulation, as an initial step this paper focuses on validation of the ITTMs ability to describe the average accumulation of liposomes across the tumor. Therefore, the results presented in this study reflect the inter-subject variations in EPR and liposome transport. The ITTM was validated by comparison to experimentally determined values for average liposome accumulation (a typical EPR metric) in two xenograft mouse tumor models and a syngeneic rabbit tumor model. When possible, the transport properties obtained through the prediction were compared with previously published measurements obtained in the same tumor model [16], [28], [29]. Additionally, the syngeneic rabbit model was used to test the ability to scale the model to larger species. Lastly, simulations were performed to understand the limitations of the ITTM and to elucidate the relationship between tumor transport properties and intra-tumoral liposome accumulation.

## Materials and Methods

### Intra-Tumoral Transport Model

The ITTM model describes convection driven trans-vascular and interstitial transport of liposomes in a solid tumor. The ITTM was based on convective transport due to the significant molecular weight of the agent (∼100 MDa) and several reports demonstrating that interstitial and transvascular diffusion is negligible compared to convection for macromolecules and liposomes [16], [17], [24], [30], [31]. The rate of accumulation of liposomes in the interstitial space of tumors is given by,
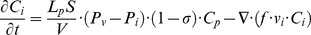
(1)


The first term on the right of equation (1) represents the trans-vascular convective flux where 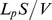 is the capillary filtration coefficient (CFC), 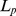 is the vascular permeability to fluid flow (hydraulic conductivity), 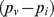 is the difference between the microvascular pressure (MVP) and IFP, 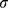
is the filtration reflection coefficient, and 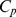 is the plasma concentration of the nanoparticle. The second term on the right represents the interstitial convective flux where 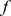
represents the fractional rate of liposome transport to fluid flow through the interstitium, 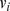
is the interstitial fluid velocity and 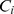 is the concentration of nanoparticles in the tumor interstitium. In this study, iodine concentration is used as a surrogate for liposome concentration due to the linear relationship between the two quantities under the assumption that the iohexol is retained within the intact liposomes [21], [22].

**Figure 1:**
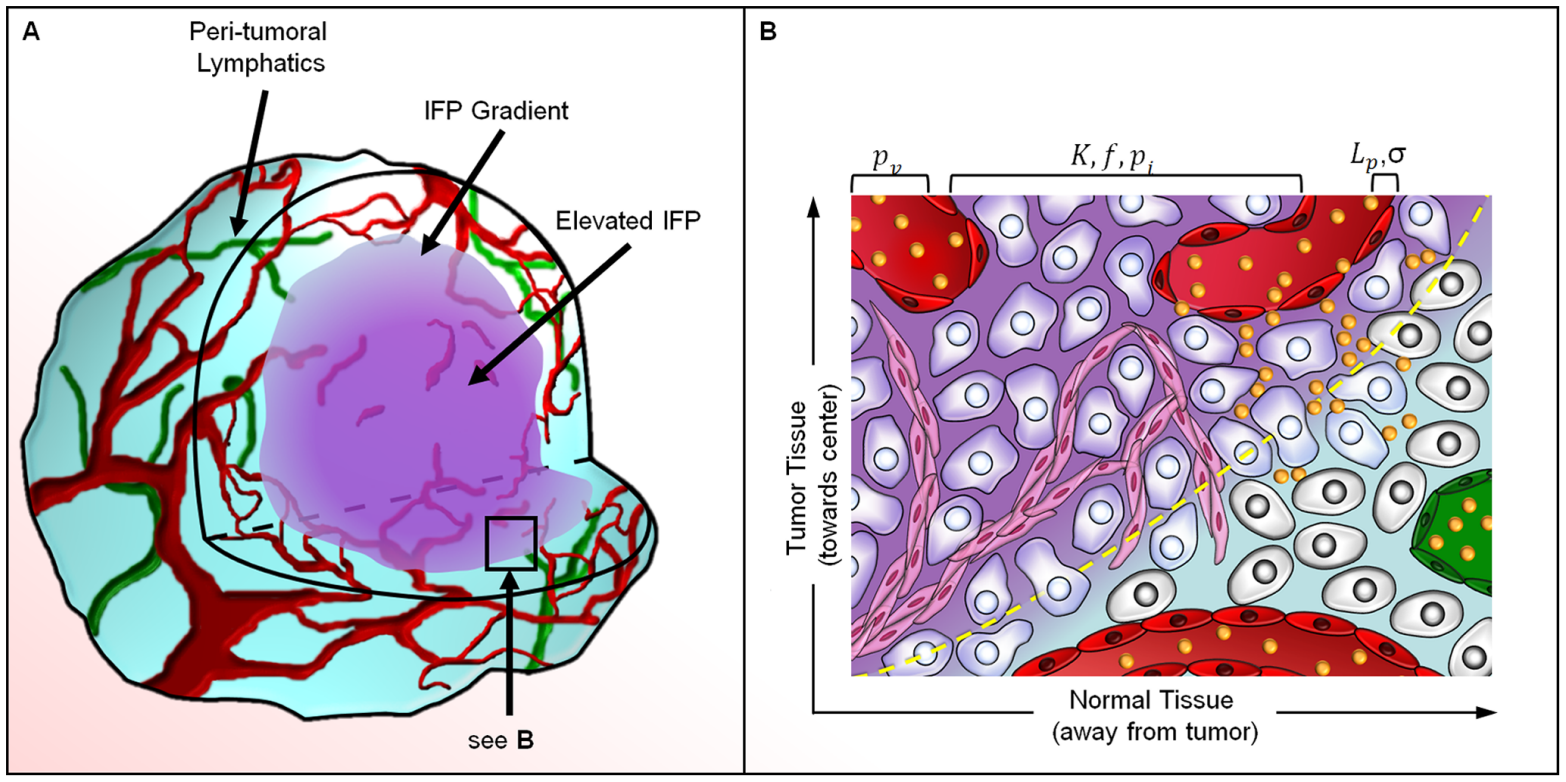
An illustration of convective transport and its relationship to the EPR effect for liposome transport. (a) Tumors experience elevated central IFP due to an increased transvascular fluid transport (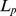), decreased interstitial fluid transport (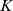), and lack of functional lymphatic vessels. Peri-tumoral lymphatics drain excess fluid at the tumor periphery, resulting in a gradient in IFP. (b) An illustration of the peri-tumoral region where the yellow dashed line indicates the border between tumor and healthy tissue. Trans-vascular (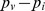) and interstitial (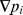) pressure gradients drive the convective transport across blood vessels and through the tumor interstitium. This process occurs predominantly along the tumor periphery where significant trans-vascular and interstitial pressure gradients are present. Convection transports liposomes through large endothelial pores (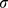) and through the extra-cellular matrix (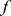) where they accumulate due to a lack of lymphatic clearance. In normal tissue, tight endothelial junctions limit liposome extravasation and functional lymphatics contribute to the clearance of the agent from the interstitium.

The parameters that define the ITTM reflect biophysical properties of the tumor microenvironment that mediate liposome transport and the EPR effect. Each of these parameters is directly or indirectly related to factors that are known to influence EPR, including: tumor blood flow, vascular organization, vascular permeability, cell density, and extracellular matrix (ECM). The ITTM and its relation to the EPR effect are illustrated in Figure 1. The principle driving force of fluid and liposome extravasation is the MVP (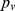), and indirectly relates both vascular organization and tumor blood flow to the rate of extravasation [15]. The rate of fluid and liposome extravasation is also determined by the vascular permeability, which are reflected in the parameters 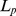 and 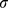, respectively. The rate of extravasation is indirectly related to the rate of tumor blood flow through 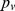.

The principle driving force for interstitial transport of liposomes is a spatial pressure gradient that can be related to interstitial fluid velocity by Darcy's law for flow through a porous medium, 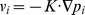. The parameter 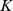
represents the interstitial permeability to fluid flow (hydraulic conductivity) and is reflective of the composition of the extra-cellular matrix (ECM) including cell density, hyaluronic acid and collagen content [16], [32]. The parameter
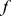
is related to ECM permeability to liposomes and reflects cell density, hyaluronic acid, collagen content, as well as the size and shape of liposomes [32]. IFP was calculated using the steady-state formula proposed in [17], 
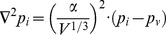
(2)where 
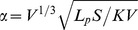
 is a unit-less number that represents the ratio of vascular to interstitial permeability to fluid flow. In general, as 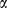 increases the IFP approaches MVP and the spatial gradient in IFP increases at the tumor periphery. The ITTM can describe tumors of arbitrary geometry and with spatio-temporally varying transport properties; however, in this study the model was simplified to a spherically symmetric tumor with uniform transport properties. Under this condition Equation 2 can be simplified to the analytic solution given in [17], with 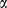 ranging from 0.5 to 150 based on previously reported tumor transport properties [29], [33], [34]. Equation (1) was solved using the finite difference method implemented in Matlab (Mathworks, Natick, MA) and the resulting solution was integrated over the tumor volume to get the average liposome (iodine) concentration as a function of time for comparison to measurements.

### Validation of the Intra-Tumoral Transport Model

Validation of the ITTM was performed by fitting the CT-based measurements of average liposome accumulation in two xenograft mouse tumor models and one syngeneic rabbit tumor model. Validation was accomplished by demonstrating that the ITTM: (1) fit the experimental data with an r^2^>0.90; (2) produced predictions of peak 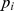 and *K* which are within the range previously reported for ME180 tumors [28], [29]; (3) can describe observed variations in the measured EPR mediated liposome accumulation in the three tumor types; and (4) can scale to larger species. The model parameters, curve fitting technique, and further details of the validation technique are provided in the supplemental information (File S1). The predicted transport properties are reported as the best-fit value and 95% CI. Statistical analysis was performed using the student t-test with a significance threshold of 0.05.

### Biophysical Modeling of the EPR Effect and its Sensitivity to Parameters

ITTM simulations were performed by varying the transport the parameters *R*, 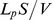, *K*, and 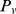 to determine their effects on tumor IFP, the average liposome accumulation, the intra-tumoral liposome distribution. The parameters were varied over a range of accepted values that are shown in Tables S1 and S4 in File S1. Further details are provided in supplemental information (File S1).

### Liposome Contrast Agent (CT-liposome) Preparation and Characterization

The liposome-based CT contrast agent was prepared according to previously described methods [21],[23]. Briefly, 1,2-dipalmitoyl-*sn*-glycero-3-phosphocholine (DPPC, MW 734) and 1,2-distearoyl-*sn*-glycero-3-phosphoethanolamine-N-poly(ethylene glycol) 2000 (DSPE-PEG_2000_, MW 2774) were purchased from Genzyme Pharmaceuticals (Cambridge, USA). Cholesterol (CH, MW 387) were obtained from Avanti Lipids Inc. (Alabaster, USA). The lipid components for the CT-liposomes (i.e. DPPC, CH, DSPE-PEG_2000_) were dissolved in anhydrous ethanol at 70°C at a molar ratio of 55∶40∶5 DPPC:CH:DSPE-PEG_2000_. Omnipaque™-300 (300 mg/mL iodine, GE healthcare, Mississauga, Canada) was added to the solution with a lipid concentration of 100 mM following ethanol removal. The final iodine concentration was approximately 45 mg mL^−1^. For the VX2 rabbit studies gadoteridol was co-encapsulated with iohexol at a concentration of 6.6 mg mL^−1^. The mean diameter of the liposomes used for all studies was approximately 80 nm, and the calculated molecular weight (MW) was ∼100 MDa. Detailed preparation and characterization procedures can be found in the supplemental information (File S1).

### Animal Models

All experiments were performed in compliance with the guidelines established by the Canadian Council on Animal Care and the Animals for Research Act of Ontario. The protocol was approved by the University Health Network Institutional Animal Care and Use Committee (Animal Use Protocol #383). Measurements of CT-liposome accumulation were performed in 3 different tumor models: (1) a human cervix carcinoma cell line (ME180) implanted orthotopically in female SCID mice (n = 4); (2) a human non-small cell lung carcinoma cell line (H520) implanted subcutaneously in male nude mice (n = 5); and (3) a syngeneic rabbit carcinoma cell line (VX2) implanted intramuscularly in male New Zealand white rabbits (n = 5). The ME180 and H520 mouse tumor models were employed, as preliminary studies by our group had shown that they result in low and high intra-tumoral liposome accumulation, respectively. The VX2 syngeneic rabbit tumor model was used to evaluate the ability to scale the ITTM to larger species. ME180 tumors were established by suturing a 2–3 mm^3^ tumor fragment onto the cervix in of female SCID mice (20–25 g) [35]. H520 tumors were established by injecting H520 cells into the subcutaneous tissue of the hind limb of female athymic nude CD-1 mice (20–25 g). VX2 tumors were established by injecting VX2 carcinoma cells obtained from 2 donor rabbits into the left lateral quadriceps of male New Zealand White rabbits (2.8–3.2 kg). The CT imaging data sets of liposome pharmacokinetics (PK) and tumor accumulation in the H520 and VX2 models were previously published in [23] and [21], respectively.

### CT Imaging of Liposome Accumulation

Longitudinal CT imaging of liposome accumulation was performed once the ME180, H520, and VX2 tumors were approximately 8.3±0.2 mm, 2.3±0.4 mm, and 21.5±4.1 mm in diameter, respectively. All animals were anaesthetized using an isoflurane-oxygen mixture. Each mouse received a bolus of 200 µL of CT-liposomes (∼0.400 mg of iodine g^−1^ and 1.20 mg of total lipid g^−1^) via the lateral tail vein. Each rabbit received a bolus of 15 mL of CT-liposomes (0.276 mg of iodine g^−1^ and 0.83 mg of total lipid g^−1^) via the marginal ear vein. CT images were acquired pre-administration and at 5 min, 1 hr, 8 hrs, 24 hrs, 48 hrs, 72 hrs, 96 hrs, 120 hrs and 144 hrs post-administration for ME180 mice. H520 tumor bearing mice underwent the same imaging protocol, with the exception of the 1 hr and 120 hr scans. VX2 tumor bearing rabbits were imaged pre-administration and 30 min, 24 hrs, 48 hrs, 72 hrs, 120 hrs, 168 hrs, 240 hrs and 336 hrs post-administration. Each ME180 mouse received a nominal CT dose of 1.7 Gy over 6 days, each H520 mouse received 1.2 Gy over 6 days, and each VX2 rabbit received a nominal dose of 135 mGy over 14 days. This dosing schedule is likely to have minimal radiation bio-effects [36]. Further details of the imaging method can be found in the supplemental information (File S1).

### Image-Based Determination of Pharmacokinetics and Tumor Accumulation

The tumor volume and descending aorta were contoured on each CT data set. The average signal intensity, in Hounsfield units (HU), was determined in each volume of interest at each time point and converted to iodine concentration (in mgI cm^−3^) using a calibration factor of 50.1±0.4 HU per mgI cm^−3^ for the mouse CT scans and 38.0±0.6HU for the rabbit CT scans. The plasma iodine concentration 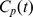 was estimated by adjusting the measured concentration in the aorta for the arterial hematocrit (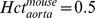, [35]) and fitting the results to a one compartment PK model. The average plasma volume fraction (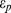) of each tumor was estimated by taking the ratio of average iodine concentration measured in the tumor to that in blood 5 min after injection for mice and 30 minutes post-injection for rabbits. At this early time point the liposomes are assumed to be predominantly intravascular. The plasma volume fraction was used to subtract the contribution of the plasma compartment from the measured iodine concentration in the tumor. The average tumor volume was determined from the contours and used to estimate an equivalent radius R, representing the radius of a sphere with a volume equal to the contoured tumor. The equivalent radius was used as input to the ITTM. Further details can be found in the supplemental information (File S1).

### Histological Analysis

Tissue sections were processed by a certified medical laboratory technologist at the Applied Molecular Profiling Laboratory (University Health Network, Toronto, ON, Canada) using standard operating procedures (SOPs). The SOPs included evaluation of negative and control sections in order to validate the positive staining of ME180 and H520 tumor tissue sections. Tumor morphology (H&E), vascularity (CD-31), perfusion (Hoechst 33342), and lymphatics (LYVE-1) were assessed in tissue sections of ME180 tumors. This provided an assessment of the transport properties that may deviate from the assumptions of the ITTM. Tumor morphology and vascularity were assessed in tissue sections of H520 tumors. Quantifiable tissue sections were not available for VX2 tumors. Analysis consisted of imaging whole tissue sections from each tumor and quantifying the percentage area of positively stained pixels. Results were compared between tumors of the same and different types. A detailed description is given in the supplemental information (File S1).

## Results

### Measurements of Liposome Accumulation

**Figure 2:**
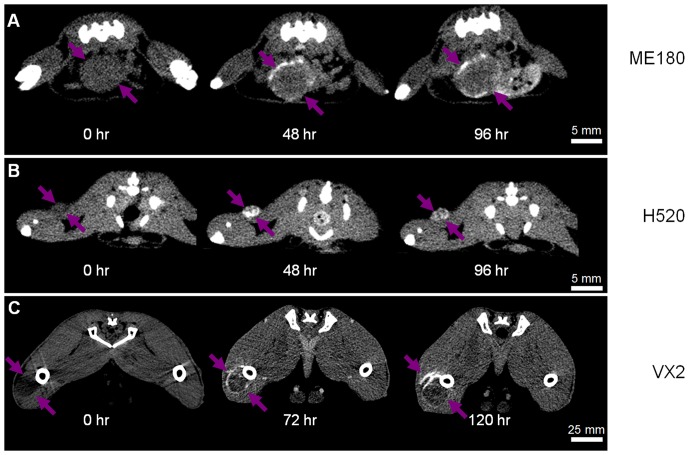
Micro-CT images of CT-liposome accumulation. Representative images of CT-Liposome accumulation are shown for an ME180 mouse orthotopic tumor (a), an H520 mouse subcutaneous tumor (b), and a VX2 rabbit intramuscular tumor (c). The arrows indicate the extent of the tumor volume. The transverse images illustrate the intra-tumoral heterogeneity of liposome accumulation; particularly in the ME180 and VX2 tumors which have predominantly peripheral liposome accumulation. Note the difference in scales.

Figure 2 shows the spatio-temporal distribution of the CT-liposomes in the ME180, H520, and VX2 tumor models. Qualitatively, the intra-tumoral distribution of liposomes was primarily along the periphery of the tumor nodules in the ME180-bearing mice and VX2-bearing rabbits. In the H520 tumors, the intra-tumoral distribution appeared heterogeneous throughout the tumor volume for the three small (<9 mm^3^) tumors and predominately peripheral in the two larger (>20 mm^3^) tumors. The tumors grew in volume from 297±20 mm^3^ to 498±26 mm^3^ in ME180 mice, from 7.1±3.2 mm^3^ to 12.6±8.7 mm^3^ in H520 mice, and from 5.7±2.9 cm^3^ to 25.0±4.7 cm^3^ in VX2 rabbits over the course of the experiments. This corresponded to an increase in equivalent radius of less than 1 mm for ME180 and H520 tumor types, which should theoretically have minimal impact on 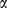, IFP, and liposome transport. The VX2 tumors increased on average by 0.7 cm, which likely impacted 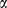, IFP, and liposome transport, but these changes were not taken into account in this study.

**Figure 3:**
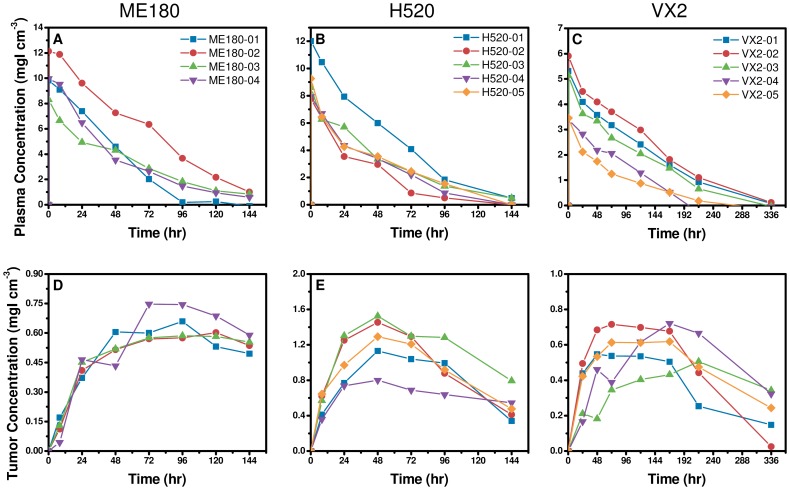
Quantification of plasma pharmacokinetics (PK) and tumor accumulation of CT-liposomes. Quantitative measures of plasma PK and average tumor accumulation is shown for ME180 mouse orthotopic tumors (a, d), H520 mouse subcutaneous tumors (b, e), and the VX2 rabbit intramuscular tumors (c, f). The plasma PK (a–c) and tumor accumulation (d–f) were obtained from the mean concentration of iodine in the blood and tumor volume, respectively. The plasma half-life of the agent was 38±9 hr, 35±6 hr, and 64±6 hr in the ME180, H520, and VX2 models, respectively. Considerable variability in rate and extent of liposome accumulation was observed between the tumor models. Error bars are smaller than the symbols.

The PK and tumor accumulation profiles of the CT-liposomes in the ME180, H520 and VX2 tumor bearing animals are shown in Figure 3. The average peak plasma concentration was 11.2±1.7 mgI cm^−3^, 8.0±1.8 mgI cm^−3^, and 5.1±1.4 mgI cm^−3^ in the ME180, H520, and VX2 tumor models, respectively. The plasma half-life of the agent was 38±9 hr, 35±6 hr, and 64±6 hr in the ME180, H520, and VX2 tumor models, respectively. The half-life of the CT-liposomes in rabbits was significantly longer compared to mice due to recognized inter-species differences (p<0.01). There was no statistically significant difference in the peak tumor accumulation of liposomes between the H520 and VX2 groups (p = 0.64). The peak liposome accumulation in tumors and the tumor area under the curve (AUC) in the ME180 group were approximately half that of the values obtained for the H520 group. Liposome accumulation curves were similar in ME180 tumors (Figure 3d); however, significant inter-subject variability was observed in H520 and VX2 tumor groups (Figures 3e and f).

### Validation of the Intra-Tumoral Transport Model

**Figure 4:**
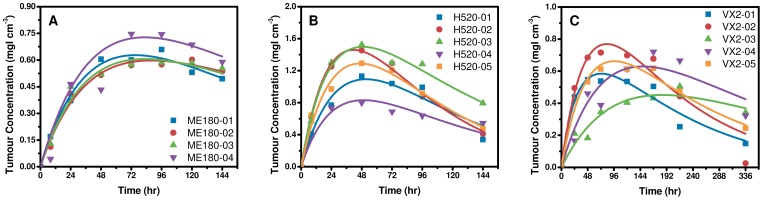
Best fit prediction of liposome accumulation . Best fit prediction of CT-liposome accumulation is show for ME180 mouse orthotopic tumors (a), H520 mouse subcutaneous tumors (c), and VX2 rabbit intramuscular tumors (d). All fits had an r^2^>0.9, with the exception of VX2-04 which had an r^2^ = 0.85. These results demonstrate that the ITTM can predict variations in liposome accumulation in different tumor types and can be scaled for use in larger species. Note the difference in scales along the axes.

The best fit tumor accumulation curves for each ME180, H520 and VX2 tumor are plotted in Figure 4. All fits had an r^2^>0.90, with the exception of VX2-04 which had an r^2^ = 0.85, when 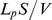, *K*, 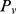 and 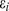 were constrained to the range of previously published independent measurements (Table S1 in File S1). This highlights that the predictions of liposome accumulation are based on realistic and independently measured transport properties in tumors. Predictions of transport parameters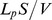, *K*, and 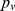 for each mouse are summarized along with their 95% CI in Table S2 in File S1 and estimates of 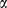, and 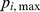 are summarized in Table S3 in File S1. The average predicted *K* and 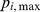
in ME180 tumors were (2.9±5.3)×10^−7^ cm^2^ mmHg^−1^ s^−1^ and 4.7±0.9 mmHg, respectively. These values were consistent with previous measurements made in the same tumor model [28], [29]. Considering each tumor individually, the 95% CIs for the best-fit values for *K* and 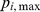
were also within the range of previously reported measurements. Additionally, the average predicted 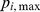 in VX2 tumors was 19.8±18.3 mmHg which overlaps with previously published measurements in the same tumor model [16]. There was no statistically significant difference in the predicted 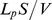, *K* and MVP between tumor types; although VX2 tumors had higher values compared to ME180 and H520 tumors. Predictions of 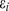 were consistent across all tumors.

On average the predicted 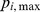 was 4.7±0.9 mmHg, 2.9±1.4 mmHg and 19.8±18.3 mmHg in ME180, H520 and VX2 tumors, respectively. There was no statistically significant difference in predictions of 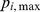 between tumor types; however, there was an observed trend of higher 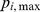 in larger tumors. The predicted 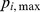 in individual tumors is consistent with the CT-liposome accumulation curves. Meaning, CT-liposome accumulation curves in ME180 tumors were similar in shape and peak accumulation, and subsequent predictions of 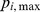
were also consistent between tumors, having a coefficient of variation of 20%. Conversely, significant variability in CT-liposome accumulation curves was observed in both the H520 and VX2 tumor models, and was reflected in predictions of
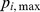
where the coefficient of variation was 48% for H520 tumors and 92% for VX2 tumors. Therefore, the ITTM suggests that inter-subject variation in CT-liposome accumulation between tumors of the same type was predominantly driven by variations in tumor IFP.

These results confirm that the ITTM is able to describe EPR mediated liposome accumulation in three different tumor models. Additionally, the ITTM can be scaled for use in larger species suggesting significant potential for clinical applicability. Finally, the ITTM attributed inter-subject variations in EPR mediated accumulation of CT-liposomes to variations in IFP between tumors.

### Histology

**Figure 5:**
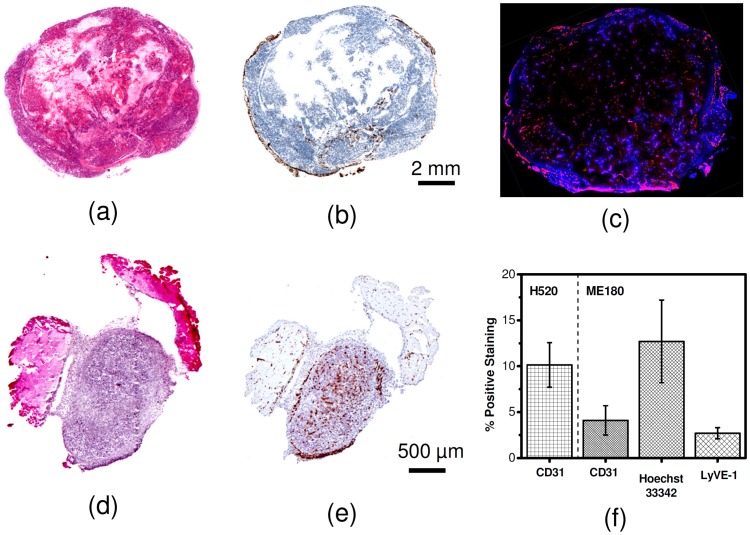
Representative histology sections for H520 and ME180 tumor tissue. (a) H&E and (b) LYVE1 sections for ME180 tumors showing patches of necrosis, and predominantly peripheral lymphatic vessels. (c) A representative section showing heterogeneous perfusion (blue) and blood vessels distribution (red) in an ME180 tumor. (d) H&E and (e) CD31 sections for H520 tumors showing no necrosis and a largely homogeneously vascularized tumor. (f) Quantitative analysis demonstrating average percent positive staining for the H520 tumor sections and ME180 tumor sections. Error bars represent the standard deviation.

ME180 tumors contain patches of necrosis scattered throughout the tumor volume (Figure 5a). The necrosis visible on ME180 sections suggests that analysis of intra-tumoral heterogeneity will be more complex for this tumor type and size. No necrosis was observed in the H520 tumor sections (Figure 5d). Lymphatic staining in ME180 tumor sections was minimal (% positive LYVE-1 was 2.7±0.6%) and limited to the periphery (Figure 5b). The ME180 tumor sections were largely avascular at the end of the study (% positive CD-31 was 4.1±1.6%) which agreed with previous findings [28]. The vascular regions were isolated to viable tissue and only 40±8% of CD-31 positive vessels were perfused (Figure 5c). The % perfused over the whole tumor section was 13±5% indicating limited perfusion at the end of the study for ME180 tumors. H520 tumor sections appeared uniformly vascularized (% positive CD-31 was 10±2%,), and due to their small size are believed to be well perfused. The H520 tumors had a twofold higher % positive CD-31 staining than ME180 tumors (p-value = 0.004), which may be a contributing factor to the two fold increase in peak tumor accumulation and tumor AUC observed from imaging.

### Biophysical Transport Modeling of the EPR effect and Sensitivity Analysis

**Figure 6:**
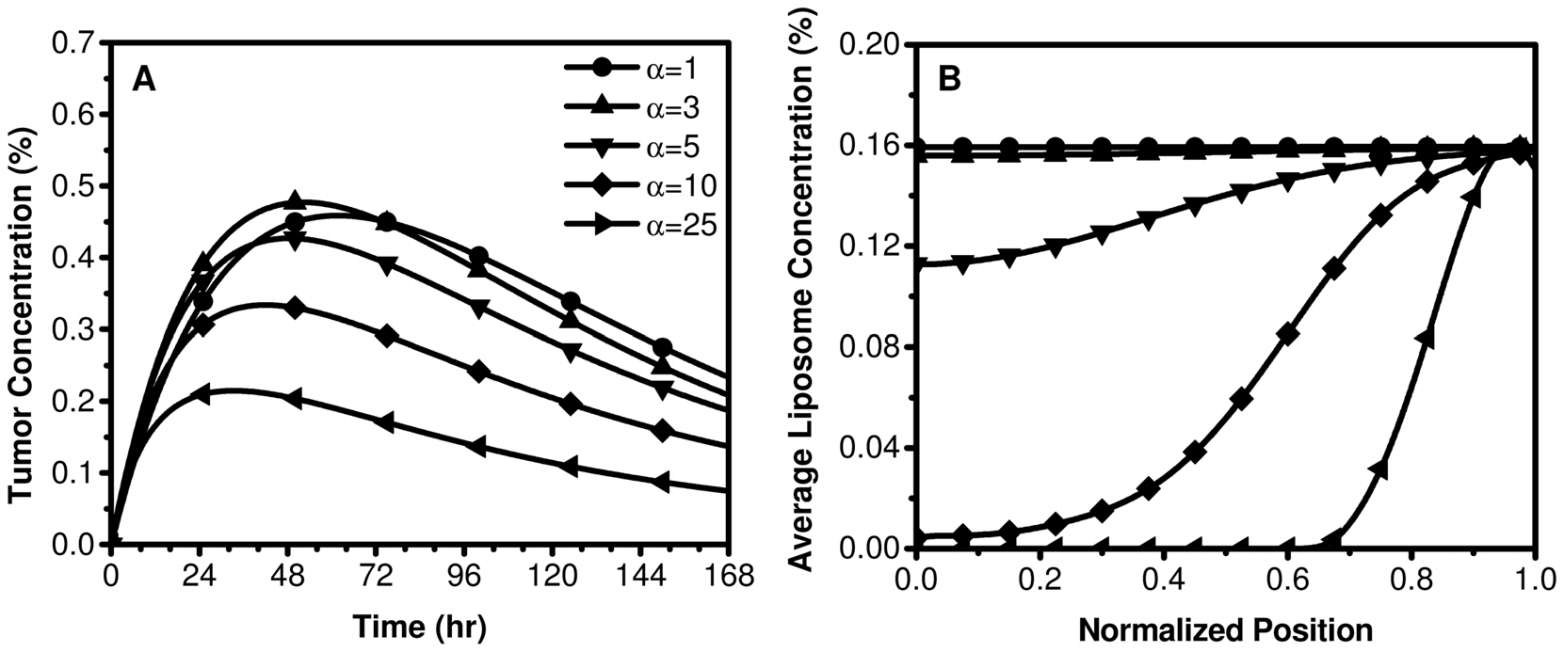
Biophysical modeling of the relationship between average liposome concentration (relative to plasma), the intra-tumoral distribution of liposomes, and tumor IFP. As α increases (which is equivalent to an increase in IFP) the ITTM model predicts a decrease in the average concentration of liposomes in the tumor volume (a) and the transitions from uniform to periphery-dominated, non-uniform intra-tumoral liposome accumulation (b).

Simulations showed that an increase in 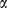 results in elevated tumor IFP in the center which drops precipitously at the periphery, and a correspondingly predominate peripheral liposome accumulation. Figure 6 and Figure S1 in File S1 demonstrate the sensitivity of the model to the biophysical transport properties (*R*,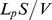, *K*, 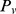) and how these parameters influence tumor IFP, the intra-tumoral accumulation of liposomes and the average liposome accumulation. The model predicts that a faster rate and higher peak in liposome accumulation occurs in low IFP tumors that have a relatively homogeneous intra-tumoral distribution of liposomes. Simulations suggest that an 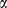
<3 is optimal for liposome therapeutics. Under these conditions, tumor IFP is consistently lower than the MVP (ratio of IFP to MVP is less than 0.9) leading to a uniform distribution of liposomes in the tumor volume.

The simulations suggest that this transport environment exists in small tumors (R<0.5 cm) or those that have a relatively low CFC (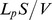
<2.5×10^−5^ mmHg^−1^ s^−1^) or high interstitial hydraulic conductivity (*K*>6.9×10^−7^ cm^2^ mmHg^−1^ s^−1^). The transition from low to high relative IFP occurs for 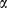
>3, and the corresponding intra-tumoral distribution of liposomes becomes predominantly peripheral. This transition can occur when either the tumor radius is larger, 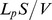 is higher, or *K* is lower than the aforementioned values. The rate of liposome accumulation and peak liposome concentration was most sensitive to tumor size and MVP. Small tumors or those with high MVP have the highest rate of liposome accumulation and peak liposome concentration, and exhibit a more homogeneous intra-tumoral liposome distribution. Increasing 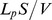 or *K* only moderately influences the shape of the liposome accumulation curves. These results suggest that a potentially advantageous strategy to improve liposome accumulation is to increase the MVP or to reduce tumor IFP by reducing tumor size, modulating the vascular permeability, or increasing the interstitial hydraulic conductivity. Indeed, several studies have demonstrated that modulating each of these parameters improves the accumulation and intra-tumoral distribution of nanoparticles [37]–[39].

## Discussion

A theoretical framework was developed to describe the transport and accumulation of liposomes in solid tumors. The ITTM is based on biophysical transport equations that describe pressure driven fluid flow across blood vessels and through the tumor interstitium. It was demonstrated that the ITTM can predict average temporal liposome accumulation in three pre-clinical tumor models with fitted parameters that reflect accepted independent measurements of the tumor microenvironment. These results highlight that the ITTM can relate the inter-subject heterogeneity in liposome accumulation to the underlying tumor transport microenvironment. Specifically, it was found that substantial inter-subject heterogeneity in liposome accumulation can be caused by variations in peak tumor IFP. Furthermore, it was demonstrated that the ITTM is scalable between species due to the ability to directly measure the plasma PK using imaging and to input this measurement into the mathematical model.

An important implication of the ITTM is its ability to relate the intra-tumoral variations in the EPR effect to the convective transport of liposomes in a solid tumor. As demonstrated in this study, the application of the ITTM to imaging of intra-tumoral liposome accumulation provides information about the spatial distribution of convective transport and tumor IFP. This has been attempted previously in a limited manner using invasive point-based mapping of radial IFP profiles in animal tumors [40]. However, this approach is not feasible in patients. Little is known about the underlying intrinsic tumor parameters that influence fluid and macromolecule flux in human malignancies. A non-invasive imaging approach to measure IFP and/or convective transport would provide valuable new clinical insight into human tumor pathophysiology and allow the prognostic and predictive effects of transport parameters to be evaluated on a large scale. For example, this approach could be used to identify convective transport factors limiting the intra-tumoral accumulation of liposomes and to identify strategies to modulate these factors to improve accumulation. However, accomplishing this requires maturing the ITTM to include the additional factors that influence intra-tumoral heterogeneity (e.g. necrosis and perfusion).

A major limitation of the study was neglecting the intra-tumoral distribution when fitting to measurements of the average CT liposome enhancement over the tumor volume. This approach was taken as an initial simple validation of the ITTM using experimental data in a manner that is consistent with previously reported EPR measurements [2]–[4], [20], [21], [23], [41]; however, it ignores the wealth of information available on intra-tumoral liposome distribution. As shown in this work, the intra-tumoral distribution of liposomes is influenced by tumor IFP; however, the underlying spatial varying characteristics of the tumor microenvironment, including: microvascular density, vascular permeability, interstitial composition and tumor necrosis also play an important role. Applying the ITTM to the spatial measurements of liposome accumulation at a single or multiple time points may provide improvements in predicting transport properties; however, fitting the ITTM to spatial measurements of liposome distribution requires incorporating measurements of spatial variations in tumor transport properties. There have been several approaches to characterize the spatially variable transport properties of tumors [42], [43]. The use of imaging techniques, such as dynamic contrast enhanced CT and diffusion weighted magnetic resonance imaging, may provide quantitative spatial measurements of tumor microenvironment properties, such as: tumor blood flow, vascular permeability, necrosis, and cell density. These independent measurements could be used as input into the ITTM and would strengthen predictions of the intra-tumoral heterogeneity in liposome transport and the EPR effect.

Moving forward, the ITTM forms the basis for transport modeling of drug delivery using imaging data. In this study a relatively high lipid dose was used for CT-liposome imaging compared to standard therapeutic doses reported for other lipid nanoparticles, such as DOXIL®, Lipoplatin™, and SPI-77. It is important to note that the ITTM compensates for alterations that lipid dose may have on PK as the image-derived measure of plasma kinetics is used as direct input to the model. There are several limitations of the presented ITTM, including: (1) accurately modeling the physico-chemical properties of liposomes in relation to trans-vascular and interstitial transport; (2) incorporating the release kinetics of the encapsulated drug; and (3) cellular uptake of liposomes by tumor cells and mononuclear phagocyte cells (MPS). Modeling of liposome properties has been limited to morphological properties that contribute to size exclusion by trans-endothelial pores and the ECM. Several other physico-chemical properties of liposomes are known to influence transport, including surface properties such as charge and hydrophilicity, as well as the presence of targeting moieties [3]. Incorporating these properties into the model is integral to understanding and optimizing the effects of liposome properties on intra-tumoral transport and predicting therapeutic response. Previous work has explored modeling the release kinetics of drugs from conventional and triggered-release liposomes, which could easily be incorporated into the ITTM and would allow for predictions of drug bioavailability using the ITTM [43]. Finally, liposomes have been shown to be internalized by tumor associated macrophages [3], which can make up a significant population in tumors [44]. Cell uptake of liposome can alter the retention kinetics of liposomes within a tumor. In the present study, cell uptake likely made a negligible contribution to variability between tumor accumulation in mice with the same tumor type; yet, it may have had a significant impact on the variability observed in tumor accumulation between tumor types. Future work will focus on integrating cell uptake into the ITTM.

CT was chosen for this study as it provides a simple tool to quantitatively assess the concentration and spatio-temporal distribution of liposomes in a solid tumor. The CT-liposome formulation used in this study is stable for weeks [22] allowing for longitudinal assessment of liposome PK and intra-tumoral accumulation. The ITTM is not restricted to CT, and several liposome formulations have been developed for MRI, PET and optical imaging [10], [22], [26]. These techniques may improve detection sensitivity allowing for administration of a decreased lipid dose of the agent, but are limited by either spatial resolution or quantitative ability. Multimodal liposomes may provide a suitable imaging platform that takes advantage of the strengths of each imaging modality [45]. For example, multimodal (e.g. optical and CT) imaging would allow for macroscopic and microscopic assessment of intra-tumoral liposome distribution in the same tumor. Combining these imaging approaches with the proposed ITTM provides a powerful tool to further understand the mechanisms that lead to intra-tumoral heterogeneity in EPR mediated liposome accumulation.

## Conclusion

We have developed a biophysical transport model to describe the total tumor and intra-tumoral accumulation of liposomes. The model, termed the intra-tumoral transport model (ITTM), was validated by comparison of predicted values to measurements of EPR mediated accumulation of liposomes in multi-species pre-clinical tumor models. The ITTM reveals the critical link between the EPR effect and IFP, and demonstrates that biophysical properties of the tumor microenvironment that influence fluid transport dynamics play an integral role in liposome accumulation. The ITTM also offers the potential for development of a quantitative, image-based approach to non-invasively estimate parameters related to IFP. Such a method could be used to guide the application of nanomedicine in a clinical setting. Applying the ITTM to the spatial measurements of liposome accumulation will enable improved predictions of transport properties, further validating the model, and bringing an image-based approach to quantitatively assess nanomedicine closer to reality. In conclusion, the ITTM provides a theoretical framework that links intra and inter-subject variations in EPR to the underlying transport properties of solid tumors.

## Supporting Information

File S1 **Supporting information file.**
(DOC)
