# Supplementary material for: A Mathematical Model of the Enhanced Permeability and Retention Effect for Liposome Transport in Solid Tumors
Source: PLoS One. 2013 Dec 2;8(12):e81157. doi: 10.1371/journal.pone.0081157 (PMC3846845; doi:10.1371/journal.pone.0081157)
Supplement: File S1 — Supporting information file. (DOC) [file pone.0081157.s001.doc]

**A Mathematical Model of the Enhanced Permeability and Retention Effect for Liposome Transport in Solid Tumors**

**File S1: Supplemental Information**

**S1. Methods and Materials**

*S1.1 CT-liposome Preparation*

1,2-dipalmitoyl-*sn*-glycero-3-phosphocholine (DPPC, MW 734) and 1,2-distearoyl-*sn*-glycero-3-phosphoethanolamine-N-poly(ethylene glycol) 2000 (DSPE-PEG_2000_, MW 2774) were purchased from Genzyme Pharmaceuticals (Cambridge, USA). Cholesterol (CH, MW 387) was obtained from Avanti Lipids Inc. (Alabaster, USA). Omnipaque™-300, a commercially available iodinated CT contrast agent that contains iohexol (M.W. 821.14) at 300 mg/mL iodine, was purchased from GE Healthcare (GE healthcare, Mississauga, Canada). The method used for preparation of the CT-liposomes was adapted from a previously published report . Lipid components for the CT-liposomes (i.e. DPPC, CH, DSPE-PEG_2000_) were dissolved in anhydrous ethanol at 70°C at a molar ratio of 55:40:5 DPPC:CH:DSPE-PEG_2000_. Omnipaque^TM^-300 was added to the solution with a lipid concentration of 100mM following ethanol removal. The solution was kept at 70°C for 4 h with intermittent vortexing. Unilamellar vesicles were formed via extrusion at 70°C using a 10-mL Lipex Extruder (Northern Lipids Inc, Vancouver, Canada) with 5 passages through two stacked 200 nm pore size Track-Etch polycarbonate membranes (Whatman Inc., Clifton, NJ) followed by 5 passages through two stacked 80 nm membranes. The unincorporated contrast agent was removed by 16 h of dialysis (MWCO 8kDa) against a 250-fold volume excess of 0.02mM HEPES-buffered saline solution (HBS, pH 7.4). The liposome formulation was then concentrated to a final iodine concentration of approximately 45 mg mL^-1^.

*S1.2 CT-Liposome Characterization*

The size distribution of the liposomes was measured by dynamic light scattering (DLS) at an angle of 90° and a temperature of 25°C using a 90Plus particle size analyzer (Brookhaven, Holtsville, NY). Samples were prepared by diluting liposome formulations with HBS to a final lipid concentration of 0.25 mM. The iodine concentration in the CT-liposomes was determined by rupturing liposomes using a 10-fold excess of ethanol, diluting in HBS, and measuring ultraviolet absorbance at 245 nm using a Cary 50 UV-visible spectrophotometer (Varian, Palo Alto, CA). The molecular weight (MW) was estimated by summing the weights of the lipids, PEG, cholesterol and iohexol molecules.

*S1.3 CT Imaging of Liposome Accumulation*

Mouse CT imaging was performed using a micro-CT system (eXplore Ultra, GE Healthcare, London, Canada). The reconstructed voxel size was 0.154x0.154x0.154 mm^3^ for the anatomical images and 0.154x0.154x0.462 mm^3^ for the DCE-CT images. Rabbit CT imaging was performed using a clinical PET/CT system (GE Discovery ST, General Electric Medical Systems, Milwaukee, WI, USA). The reconstructed voxel size was 0.430x0.430x0.625 mm^3^. CT Liposome injections were performed using an injected pump (Harvard Apparatus, Canada) and a 27G butterfly catheter inserted into the lateral tail vein for M180 tumor bearing mice. Injections were performed manually for H520 tumor bearing mice and VX2 tumor bearing rabbits.

*S1.4 Measurements of Liposome Pharmacokinetics and Accumulation*

The CT data sets for each time point and mouse were imported into MicroView 2.2 (GE Healthcare) and three-dimensional volumes of interest analysis for the tumor and descending aorta were generated. The contours were made using a semi-automatic approach that combined manual contouring and threshold refinement. The tumor volume of interest was used to estimate an equivalent radius R, representing the radius of a sphere with an equivalent volume, at each time point. The equivalent radius was then averaged over time and used as input to the ITTM (Eqns. 1, 2 and S1). The signal enhancement in the tumor and descending aorta were estimated by computing the temporal change in the mean HU in each compartment relative to the mean HU measured pre-liposome administration. The iodine concentration (in mgI cm^-3^) was determined by scaling the relative signal enhancement by a calibration factor of 50.1±0.4 HU per mgI cm^-3^ for the mouse CT scans and 38.0±0.6HU for the rabbit CT scans. The calibration factor was obtained by CT scanning 5 syringes with increasing concentrations (1.4, 2.8, 5.6, 11.3, 22.5, and 45 mgI mL^-1^) of the CT-liposomes and determining the slope of a HU vs CT-Liposome concentration curve.

The plasma pharmacokinetics (PK) for each mouse were estimated by fitting the plasma concentration to a one compartment PK model and used as input to the ITTM (Eqn. 1). The average plasma volume fraction ($\varepsilon_{p}$) of each tumor was estimated by taking the ratio of average iodine concentration measured in the tumor to that in blood 5 min after injection in mice and 30 min in rabbits. The plasma volume fraction was used to subtract the contribution of the plasma compartment from the measured iodine concentration in the tumor, leaving an estimate of the apparent interstitial concentration (referred to as tumor concentration in Figures 3 and 4). That is , where the subscript *t,* *i* and *p* denote the tumor, interstitial and plasma compartments. The plasma blood pool concentration is given by where $\text{Hct}_{a}=0.5$ based on previous estimations . The ITTM described in this paper was fit to the measurement of $C_{i}(t)$, where $\varepsilon_{i}$ was a free parameter determined by the curve fitting process.

*S1.5 Histological Analysis*

Tumor morphology, vascularity, perfusion, and lymphatics were assessed in each ME180 tumor bearing mouse. Tumor perfusion was assessed at the end of the experiment by i.v. injection of the fluorescent dye Hoechst 33342 (40 mg kg^-1^ dissolved in PBS, Sigma-Aldrich, MW=561.93 Da) immediately following the last imaging time point. Sixty seconds after injection the animal was sacrificed by cervical dislocation and the tumors were surgically resected. Each tumor was divided into two equal hemispheres along a plane parallel to the CT imaging slice. One hemisphere was fixed in 10% formalin for 48 hours, dehydrated in 70% ethanol, mounted in paraffin and then cut into 4 µm sections. The second hemisphere was rapidly frozen over liquid nitrogen in Tissue-Tek optimum cutting temperature compound (Sakura Finetek Inc., Torrance, CA, USA) and cut into 5 µm sections. The paraffin-embedded tissue was stained for morphology using H&E, blood vessels using goat derived anti-mouse CD31 and for lymphatics using rabbit derived anti-mouse LYVE-1 (1/200 Abcam, Canada). The frozen sections were stained for blood vessels using a rat derived anti-mouse CD31 and cyanine-3 (Cy3)-donkey anti-rat secondary. Tumor morphology and vascularity for H520 tumors was assessed in rapidly frozen tissue sections that were stained for morphology using haematoxylin and eosin (H&E) and blood vessels using goat derived anti-mouse CD31.

The stained paraffin-embedded sections were imaged in their entirety using a digital microscope (ScanScope CD, Aperio Technologies, Vista, CA, USA) and 40x magnification. For H520 tumor sections the total area of positive CD-31 staining was quantified using the positive pixel algorithm provided by the Aperio analysis software ScanScope. The same technique was used to quantify the total area of positive lymphatic staining in ME180 tissue. The settings were standardized for each section and the % positive staining was assessed by taking the ratio of positive pixels to the total number of pixels in the tissue section. The stained frozen ME180 tumor sections were imaged in their entirety using a wide field fluorescent tiling microscope (Olympus BX50, Centre Valley, PA, USA) with a 10x objective (N.A. =0.4, W.D.=3.1, F.N. 26.5, Olympus, USA). Separate images were obtained for Hoechst 33342 staining and CD31 staining. Analyses were performed in Matlab 2009b (Mathworks Inc., Natick, MA) and the % perfused area and the % vascular area were calculated by taking the ratio of the number of positively stained pixels to the total number of pixels in the image. Additionally, the % perfused vasculature was calculated by taking the ratio of the number of positively staining CD31 pixels which co-localized with positively stained Hoechst 33342 pixels to the total number of pixels in the image.

**S2. Curve Fitting of CT-Liposome Measurements**

*S2.1 Curve Fitting and Analysis*

The prediction of the average accumulation of liposomes was calculated by integrating the solution to Equation 1 over the tumor volume, giving:

(S1)

In the case of spherical symmetry, the interstitial concentration in Equation 1 is given by$C_{i}(r,t)$, where r is the radial position. Equation S1 was fit to the measured average liposome accumulation curves (Figure 2 d-f) using a nonlinear least-squares minimization method (Matlab 2009a, Mathworks, Natick, MA). The fitted (i.e. free) parameters were $L_{p},K,p_{v}$ and$\varepsilon_{i}$with the remainder of the ITTM parameters fixed (Table S1). The free parameters were constrained within the range of previously published values. An attempt to find the global minimum of the least-squares objective function was performed by using multiple initial conditions in the fitting process. The initial conditions were chosen by stepping evenly through the range of transport parameters. The coefficient of determination (r^2^) was used as the goodness of fit metric and the solution with the lowest residuals was considered the best fit. The 95% confidence interval of each free parameter was assessed using the Monte Carlo Method described by Motulsky and Christopoulos .

| **Parameters** | **Tissue** | **Type** | **Bounds \ Value** | **References** |
| --- | --- | --- | --- | --- |
|  | Tumor | Free | 1*x*10^-7^ to 1*x*10^-3^ |  |
|  | Normal | Fixed | 2.5*x*10^-6^ |  |
|  | Tumor | Free | 1*x*10^-8^ to 1*x*10^-4^ |  |
|  | Normal | Fixed | 8.53*x*10^-9^ |  |
|  | Tumor | Free | 5 to 50 |  |
|  | Tumor | Free | 0.1 to 0.6 |  |
|  | Tumor | Fixed | 0.19 |  |
|  | Tumor | Fixed | 0.5 |  |
| $R(\text{cm})$ | Tumor | Fixed | Measured using CT |  |
|  | Tumor | Fixed | Measured using CT |  |

**Table S1:** *Parameters used in the ITTM. The transvascular reflection coefficient $\sigma$was estimated using cylindrical pore theory with an estimated microvascular pore diameter of 300nm . The interstitial retardation coefficient* $f$ *was estimated using the empirical law developed by Swabb et al. using a tissue glycosaminoglycan concentration of 0.01 g/100 g wet tissue and a solute MW of 100MDa.*

*S2.2 Results of fitting ITTM to Liposome Accumulation Data*

The best fit parameters and the estimated $\alpha$ and$p_{i\text{,max}}$ for each mouse tumor are given in Table S2 and Table S3 respectively. There was a high variability and large 95% CI for each parameter between tumors of the same type and difference types. As such there is no statistically significant difference was found between any of the transport properties. The variability for a given transport parameter between the individual mice and rabbits may be reflective of the underlying biological variability; however, the large confidence interval suggest the dominant issue was insufficient measurement information to precisely estimate the transport parameters. The high variability and large 95% CI are likely reflective of not accounting for the spatio-temporal heterogeneity in transport microenvironment and the limited degrees of freedom (DOF) in the fitting process. Taking measurements at more time points would provide a better representation of the shape of the liposome accumulation curves, and may improve the precision of predicted parameters; however, issues with necrosis and changes in vascular morphology, permeability, perfusion, etc. over time will affect the accuracy of predictions. A potentially more robust method would be to fit Equation 1 to the intra-tumoral distribution of liposomes at a given time point. The high spatial resolution of CT imaging provides ample data for fitting, and more importantly, as shown in Figure 6, the spatial distribution of liposomes is directly related to the spatial distribution of tumor IFP. Due to the distinct relationship between tumor IFP and liposome accumulation, fitting the spatial distribution may provide a more precise estimate of transport properties. In addition to increasing the precision of predictions, this method would allow for estimates of transport parameters, such as IFP, at multiple time points. The challenge, however, remains accounting for other factor which influence the intra-tumoral transport of liposomes such as: heterogeneity in vascular density, vascular permeability, blood flow, and tumor necrosis. Currently, the ITTM assumes the transport microenvironment is a uniform and static system, when in reality there are spatio-temporal variations in $L_{p}S/V$,$p_{v}$, $\sigma$, $f$, and$K$.

|  |  |  |  |  |
| --- | --- | --- | --- | --- |
| ME180-01 | 25.1 (19.5, 35.7) | 0.35 (0.21, 5.89) | 5.0 (5.0, 5.3) | 0.25 (0.18, 0.27) |
| ME180-02 | 46.5 (37.2, 63.1) | 0.15 (0.12, 0.34) | 5.0 (5.0, 5.3) | 0.17 (0.14, 0.18) |
| ME180-03 | 28.1 (20.6, 47.2) | 0.15 (0.12, 1.75) | 5.8 (5.2, 6.5) | 0.28 (0.18, 0.30) |
| ME180-04 | 6.3 (2.05, 19.9) | 10.8 (0.48, 824) | 9.2 (6.4, 12.6) | 0.23 (0.19,0.33) |
| Average | 26.5±16.4 | 2.9±5.3 | 6.2±2.0 | 0.23±0.05 |
|  |  |  |  |  |
| H520-01 | 13.5 (9.5, 20.0) | 995 (2, 1000) | 11.2 (8.4, 14.1) | 0.13 (0.12, 0.14) |
| H520-02 | 11.7 (9.5, 13.5) | 999 (31, 1000) | 11.9 (10.7, 14.0) | 0.37 (0.36, 0.38) |
| H520-03 | 93.4 (68.2, 115) | 0.12 (0.1, 0.4) | 5.2 (5.0, 5.4) | 0.35 (0.30, 0.36) |
| H520-04 | 46.9 (27.8, 70.6) | 0.13 (0.1, 6.8) | 5.1 (5.0, 5.7) | 0.22 (0.19, 0.24) |
| H520-05 | 28.4 (22.0, 36.7) | 249 (3.28, 1000) | 5.6 (5.0, 6.5) | 0.27 (0.25, 0.28) |
| Average | 38.8±33.6 | 448±510 | 7.8±3.5 | 0.27±0.10 |
|  |  |  |  |  |
| VX2-01 | 4405 (748, 8434) | 651 (417, 959) | 42.4 (35.4, 50.0) | 0.13 (0.12, 0.15) |
| VX2-02 | 70.8 (8.7, 6605) | 998 (200, 1000) | 38.1 (32.3, 50.0) | 0.14 (0.13, 0.17) |
| VX2-03 | 1.4 (1.0, 2.1) | 541 (23, 1000) | 17.5 (14.0.,19.7) | 0.26 (0.22, 0.31) |
| VX2-04 | 1.1 (1.0, 1.6) | 988 (19, 1000) | 26.3 (20.2, 29.4) | 0.50 (0.43, 0.56) |
| VX2-05 | 963 (287, 1839) | 133 (70, 540) | 9.8 (8.7, 11.0) | 0.54 (0.47, 0.60) |
| Average | 1088±1898 | 662±358 | 26.8±13.7 | 0.31±0.19 |

**Table S2:** *Best fit parameters obtained by fitting the ITTM to the tumor accumulation curves measured in each mouse. The 95% CI of the best fit predictions are shown in brackets. The average and standard deviation for each parameter was calculated using the best fit values.*

|  | (95% CI) | (95% CI) |
| --- | --- | --- |
| ME180-01 | 3.8 (1.0, 4.8) | 4.7 (3.8, 5.0) |
| ME180-02 | 7.8 (5.3, 8.9) | 5.0 (4.9, 5.2) |
| ME180-03 | 5.9 (1.8, 7.1) | 5.7 (4.8, 6.1) |
| ME180-04 | 0.35 (0.03, 1.73) | 3.5 (2.0, 5.4) |
| Average | 4.5±3.2 | 4.6±0.8 |
|  |  |  |
| H520-01 | 0.01 (0.01, 0.33) | 1.9 (1.6, 2.3) |
| H520-02 | 0.01 (0.01, 0.08) | 2.3 (2.1, 2.4) |
| H520-03 | 4.13 (2.14, 4.72) | 4.7 (4.1, 5.1) |
| H520-04 | 2.82 (0.10, 3.67) | 4.1 (2.1, 4.6) |
| H520-05 | 0.03 (0.02, 0.29) | 1.7 (1.5, 1.9) |
| Average | 1.4±1.9 | 3.0±1.4 |
|  |  |  |
| VX2-01 | 3.55 (1.22, 5.85) | 42.4 (35.4, 50.0) |
| VX2-02 | 0.40 (0.14, 5.64) | 36.6 (30.4, 50.0) |
| VX2-03 | 0.06 (0.04, 0.33) | 4.56 (3.54, 5.72) |
| VX2-04 | 0.04 (0.04, 0.28) | 5.58 (4.59, 7.17) |
| VX2-05 | 3.15 (1.11, 4.73) | 9.81 (8.66, 10.89) |
| Average | 1.44±1.76 | 19.8±18.2 |

**Table S3:** *The transport* *properties* $\alpha$ *and the maximum IFP ($p_{i\text{,max}}$) were calculated using the best fit values from given in Table S2. The 95% CI are shown in brackets. The average and standard deviation for each tumor type was also determined from the best-fit values. There is no statistically significant difference between the averaged parameters when comparing ME180, H520 and VX2 tumors.*

**S3. Biophysical Transport Modeling of the EPR Effect and Sensitivity Analysis**

*S3.1 Methods:*

Biophysical transport modeling simulations were performed by varying the parameters, *R*,$L_{p}S/V$, *K*, and $p_{v}$, in order to elucidate their effects on IFP, the intra-tumoral distribution of liposomes and average liposome accumulation. The range of values for each parameter is given in Table S3. The nominal values for $L_{p}S/V$, *K*, and$p_{v}$were chosen based on previously published data. Each parameter was changed while fixing the remaining at their nominal value. For *R*, $L_{p}S/V$, and *K* the range of values were chosen to produce the same $\alpha$.

| **Parameter** | **Nominal Value** | **Range for Simulations** | **Alpha (s)** |
| --- | --- | --- | --- |
| $R(cm)$ | 0.5 | 0.17 to 4.2 | 1 to 25 |
| $\frac{L_{p}S}{V}(mmHg^{-1}\cdot s^{-1})$ | 2.5*x*10^-5^ | (0.28 to 180) *x* 10^-5^ |  |
| $K(cm^{2}\cdot mmHg^{-1}\cdot s^{-1})$ | 7.0*x*10^-7^ | (63 to 0.1) *x* 10^-7^ |  |
| $p_{v}(mmHg)$ | 10 | 5 to 30 | 3 |

**Table S4:** Values used for the biophysical transport modeling and sensitivity analysis.

*S3.2 Results:*

The ITTM simulations demonstrated how the underlying biophysical transport properties (*R*, $L_{p}S/V$, *K*, $p_{v}$) influences tumor IFP, the average liposome accumulation, and the intra-tumoral liposome distribution, and (Figure S1)**.** In general, the simulations showed that as $\alpha$increases, tumor IFP increases, the rate of accumulation and peak liposome concentration decreases, and the intra-tumoral distribution of liposomes become predominantly peripheral. The ITTM predictions of liposome accumulation is most sensitive to the transport properties *R* and $p_{v}$. These results indicate that it is more efficacious to treat tumors with lower IFP. Given that most solid tumors have elevated IFP, efficient liposome drug delivery would benefit from pre-treating tumors with agents that lower tumor IFP. As the biophysical transport modeling indicates this would improve the rate of accumulation, peak accumulation and intra-tumoral distribution of liposomes. Some studies have shown that modifiers such as vasoactive agents , molecularly targeted agents , and radiation therapy can reduce tumor IFP and improve the delivery of macromolecules to the solid tumors. While, these studies did not look at improvement in intra-tumor distribution, the reported mechanism of action in reducing IFP is in line with the ITTM framework presented in this study .

|  | **Average Tumor**  **Accumulation** | **Intra-tumoral Liposome**  **Distribution at 48 hrs** |
| --- | --- | --- |
|  |  |  |
|  |  |  |
|  |  |  |
|  |  |  |

**Figure S1:** Biophysical modeling results which demonstrates how the underlying transport properties (*R*,$L_{p}S/V$, *K*, and $p_{v}$) of the tumor influences tumor IFP, average liposome accumulation, and the intra-tumoral distribution of liposomes**.** For *R*, $L_{p}S/V$, and *K* the symbols denote different $\alpha$values obtained by varying the respective parameter while keeping the remaining parameters fixed (see Table S4). For $p_{v}$ the symbols denote different MVPs. The y-axis represents the % of liposomes which have accumulated in the tumor, relative to the initial plasma concentration, and is an average over the tumor volume (column 1) or at a given spatial location within the tumor (column 2).

**References**

1. Dunne M, Zheng J, Rosenblat J, Jaffray DA, Allen C (2011) APN/CD13-targeting as a strategy to alter the tumor accumulation of liposomes. Journal of Controlled Release 154: 298-305.

2. Zheng J, Jaffray D, Allen C (2009) Quantitative CT imaging of the spatial and temporal distribution of liposomes in a rabbit tumor model. Molecular Pharmaceutics 6: 571-580.

3. Cairns RA, Hill RP (2004) A fluorescent orthotopic model of metastatic cervical carcinoma. Clinical and Experimental Metastasis 21: 275-282.

4. Motulsky H, Christopoulos A (2004) Fitting models to biological data using linear and nonlinear regression: a practical guide to curve fitting: Oxford University Press, USA.

5. Milosevic M, Lunt SJ, Leung E, Skliarenko J, Shaw P, et al. (2008) Interstitial permeability and elasticity in human cervix cancer. Microvascular Research 75: 381-390.

6. Baxter LT, Jain RK (1989) Transport of fluid and macromolecules in tumors. I. Role of interstitial pressure and convection. Microvascular research 37: 77-104.

7. Sevick EM, Jain RK (1991) Measurement of capillary filtration coefficient in a solid tumor. Cancer Research 51: 1352.

8. Jain RK (1988) Determinants of tumor blood flow: a review. Cancer research 48: 2641-2658.

9. Anderson JL, Malone DM (1974) Mechanism of osmotic flow in porous membranes. Biophysical Journal 14: 957-982.

10. Swabb EA, Wei J, Gullino PM (1974) Diffusion and convection in normal and neoplastic tissues. Cancer Research 34: 2814.

11. Hobbs SK, Monsky WL, Yuan F, Roberts WG, Griffith L, et al. (1998) Regulation of transport pathways in tumor vessels: role of tumor type and microenvironment. Proceedings of the National Academy of Sciences 95: 4607.

12. Seki T, Fang J, Maeda H (2009) Enhanced delivery of macromolecular antitumor drugs to tumors by nitroglycerin application. Cancer Science 100: 2426-2430.

13. Zlotecki RA, Baxter LT, Boucher Y, Jain RK (1995) Pharmacologic modification of tumor blood flow and interstitial fluid pressure in a human tumor xenograft: network analysis and mechanistic interpretation. Microvascular research 50: 429-443.

14. Zlotecki RA, Boucher Y, Lee I, Baxter LT, Jain RK (1993) Effect of angiotensin II induced hypertension on tumor blood flow and interstitial fluid pressure. Cancer research 53: 2466.

15. Tong RT, Boucher Y, Kozin SV, Winkler F, Hicklin DJ, et al. (2004) Vascular normalization by vascular endothelial growth factor receptor 2 blockade induces a pressure gradient across the vasculature and improves drug penetration in tumors. Cancer research 64: 3731.

16. Vlahovic G, Ponce AM, Rabbani Z, Salahuddin FK, Zgonjanin L, et al. (2007) Treatment with imatinib improves drug delivery and efficacy in NSCLC xenografts. British Journal of Cancer 97: 735-740.

17. Znati CA, Rosenstein M, Boucher Y, Epperly MW, Bloomer WD, et al. (1996) Effect of radiation on interstitial fluid pressure and oxygenation in a human tumor xenograft. Cancer research 56: 964.

18. Jain RK, Tong RT, Munn LL (2007) Effect of vascular normalization by antiangiogenic therapy on interstitial hypertension, peritumor edema, and lymphatic metastasis: insights from a mathematical model. Cancer Research 67: 2729-2735.
